# Predictors of all-cause and cardiovascular disease mortality in type 2 diabetes: Diabetes Heart Study

**DOI:** 10.1186/s13098-015-0055-y

**Published:** 2015-06-28

**Authors:** Laura M. Raffield, Fang-Chi Hsu, Amanda J. Cox, J. Jeffrey Carr, Barry I. Freedman, Donald W. Bowden

**Affiliations:** Molecular Genetics and Genomics Program, Wake Forest School of Medicine, Winston-Salem, NC USA; Center for Human Genomics, Wake Forest School of Medicine, Winston-Salem, NC USA; Center for Diabetes Research, Wake Forest School of Medicine, Winston-Salem, NC USA; Department of Biostatistical Sciences, Wake Forest School of Medicine, Winston-Salem, NC USA; Department of Biochemistry, Wake Forest School of Medicine, Winston-Salem, NC USA; Department of Radiology, Vanderbilt University Medical Center, Nashville, TN USA; Department of Internal Medicine - Nephrology, Wake Forest School of Medicine, Winston-Salem, NC USA; Center for Genomics and Personalized Medicine Research, Wake Forest School of Medicine, Medical Center Boulevard, Winston-Salem, NC 27157 USA

**Keywords:** Type 2 diabetes, Mortality, Coronary artery calcified plaque, Urine albumin:creatinine ratio

## Abstract

**Background:**

Many studies evaluated the best predictors for cardiovascular disease (CVD) events in individuals with type 2 diabetes (T2D), but few studies examined the factors most strongly associated with mortality in T2D. The Diabetes Heart Study (DHS), an intensively phenotyped family-based cohort enriched for T2D, provided an opportunity to address this question.

**Methods:**

Associations with mortality were examined in 1022 European Americans affected by T2D from 476 DHS families. All-cause mortality was 31.2 % over an average 9.6 years of follow-up. Cox proportional hazards models with sandwich-based variance estimation were used to evaluate associations between all-cause and CVD mortality and 24 demographic and clinical factors, including coronary artery calcified plaque (CAC), carotid artery intima-media thickness, medications, body mass index, waist hip ratio, lipids, blood pressure, kidney function, QT interval, educational attainment, and glycemic control. Nominally significant factors (*p* < 0.25) from univariate analyses were included in model selection (backward elimination, forward selection, and stepwise selection). Age and sex were included in all models.

**Results:**

The all-cause mortality model selected from the full DHS sample included age, sex, CAC, urine albumin: creatinine ratio (UACR), insulin use, current smoking, and educational attainment. The CVD mortality model selected from the full sample included age, sex, CAC, UACR, triglycerides, and history of CVD events. Beyond age, the most significant associations for both mortality models were CAC (2.03 × 10^−4^ ≤ *p* ≤ 0.001) and UACR (1.99 × 10^−8^ ≤ *p* ≤ 2.23 × 10^−8^). To confirm the validity of the main predictors identified with model selection using the full sample, a two-fold cross-validation approach was used, and similar results were observed.

**Conclusions:**

This analysis highlights important demographic and clinical factors, notably CAC and albuminuria, which predict mortality in the general population of patients with T2D.

**Electronic supplementary material:**

The online version of this article (doi:10.1186/s13098-015-0055-y) contains supplementary material, which is available to authorized users.

## Background

The Diabetes Heart Study (DHS) is an ongoing family-based cohort study investigating the epidemiology and genetics of cardiovascular disease (CVD) in a population-based sample. Prior analyses in this type 2 diabetes (T2D)-enriched cohort [1] have individually examined contributors to all-cause and CVD mortality. Coronary artery calcified atherosclerotic plaque (CAC), a measure of subclinical CVD [2, 3], C-reactive protein (CRP) [4], biventricular volume [5], heart rate-corrected electrocardiographic QT interval [6], serum albumin and creatine, estimated glomerular filtration rate (eGFR), and urine albumin:creatinine ratio (UACR) [7] were all found to predict mortality in the DHS.

Prior studies have examined risk prediction scores for CVD events in patients with T2D [8, 9], which is an important contributor to mortality [10]. However, fewer studies have attempted to evaluate the best predictors of all-cause and CVD mortality in T2D [11–14]. We performed a comprehensive analysis of which factors were the strongest independent predictors of all-cause and CVD mortality using model selection (backward elimination, forward selection, and stepwise selection) in European Americans (EAs) with T2D from the DHS.

## Methods

### Study design and sample

The DHS recruited T2D-affected siblings without advanced renal insufficiency from 1998 through 2005 in western North Carolina. T2D was defined as diabetes developing after the age of 35 years initially treated with changes in diet and exercise and/or oral agents, in the absence of historical evidence of ketoacidosis or initial treatment with insulin. Fasting glucose and glycated hemoglobin (HbA_1c_) concentrations were assessed at the exam visit. Ascertainment and recruitment criteria have been described [1]. DHS participants were recruited from the general population and broadly reflect the demography of T2D in our community. Importantly, prior evidence of CVD was not an exclusion to participate. All study protocols were approved by the Institutional Review Board at Wake Forest School of Medicine, and all participants provided written informed consent.

Participant examinations were conducted in the General Clinical Research Center of Wake Forest Baptist Medical Center. Examinations included interviews for medical history and health behaviors, anthropometric measures, resting blood pressure, electrocardiography, fasting blood sampling for laboratory analyses, and spot urine collection. Low-density lipoprotein (LDL) cholesterol was calculated using the Friedewald equation, and LDL measures were considered valid for subjects whose triglycerides were less than 400 mg/dL. Estimated glomerular filtration rate (eGFR) was computed using the CKD-EPI equation [15]. CAC was assessed using computed tomography (CT), summing the left main, left anterior descending, circumflex, posterior descending, and right coronary arteries. CT scans were performed on multi-detector CT scanners with cardiac gating in chest scans. CAC scores were measured as previously described and validated [16, 17]. Data on prior CVD events was self-reported by participants and non-adjudicated. Individuals were considered to have a history of prior CVD if they self-reported prior MI, angina, stroke, or vascular procedures including coronary angioplasty, coronary artery bypass graft, or endarterectomy, or if they had Q wave abnormalities indicative of prior MI.

Mortality was assessed using the National Social Security Death Index. For deceased participants, length of follow-up was determined from the date of initial study visit to date of death. For all other participants the length of follow-up was determined from the date of the initial study visit to December 31, 2013. When possible, copies of death certificates were obtained from county or state Vital Records Offices to determine cause of death. Both all-cause and CVD mortality were analyzed; cause of death was categorized based on death certificates as CVD mortality (myocardial infarction, congestive heart failure, cardiac arrhythmia, sudden cardiac death, peripheral vascular disease, and stroke) or as mortality from other causes. For 18 patients, cause of death information could not be obtained, so these participants were excluded from analyses of CVD mortality.

### Statistical methods

Cox proportional hazards models with sandwich-based variance estimation (due to the family structure of the DHS) in SAS 9.3 were used to evaluate associations with mortality. For model building, 24 potential predictors of mortality for which data was available for most individuals in the cohort, including CAC, carotid artery intima media thickness (IMT), medications, body mass index (BMI), waist hip ratio (WHR), lipids, blood pressure, kidney function measures, electrocardiographic QT interval, educational attainment, and glycemic control measures, were evaluated in separate models for their association with all-cause mortality and CVD mortality. Variables were transformed prior to analysis; the square root of BMI and high-density lipoprotein (HDL) cholesterol, the square of WHR, and the natural log of glucose, HbA_1c_, CAC, total cholesterol, triglycerides, UACR, QT interval, diabetes duration, IMT, and mean arterial pressure were used. Nominally significant factors (*p* < 0.25) from univariate analyses were included in model selection performed in SAS 9.3. We confirmed that no factors included in model selection were strongly correlated (*r* > 0.8). Age and sex were included in all models. For backward elimination selection, factors with a *p*-value < 0.05 for association with mortality were retained in the model. For stepwise selection, factors meeting a threshold of a *p*-value < 0.25 could enter into the model but required a *p*-value < 0.05 to be retained. For forward selection, factors meeting a threshold of a *p*-value < 0.05 could enter into the model. In our analysis, results from the multiple Cox regression models were concordant for forward, backward, and stepwise model selections. Calculations for area under the receiver operating characteristic curve (AUC) were performed in STATA version 12.1.

## Results

All self-described EAs from the DHS with T2D (1022 individuals from 476 families) were included in the analyses. Table 1 displays their demographic and clinical characteristics, presented for the full sample and stratified by mortality status. For the full cohort, mean ± standard deviation (SD) diabetes duration was 10.4 ± 7.2 years. Prevalence of hypertension, obesity, subclinical CVD based on CAC, and prior CVD events were high. Over a mean ± SD follow-up of 9.6 ± 3.2 years, all-cause mortality was 31.2 % and CVD mortality was 14.3 %.

**Table 1:** Demographic and clinical characteristics of European American participants with type 2 diabetes, stratified by mortality status

	Full Sample (*n* = 1022)		Living (*n* = 703)		Deceased (*n* = 319)	
Trait	Mean (SD) or %	Median (range)	Mean (SD) or %	Median (range)	Mean (SD) or %	Median (range)
Age (years)	62.43 (9.07)	62.80 (34.21, 85.98)	60.64 (8.48)	60.54 (34.21, 81.77)	66.36 (9.09)	67.03 (34.34, 85.98)
Female Sex (%)	51.5 %		54.8 %		44.2 %	
Current Smoking (%)	16.1 %		14.0 %		20.8 %	
Past Smoking (%)	43.4 %		43.6 %		43.1 %	
History of Cardiovascular Disease (%)	43.2 %		37.3 %		56.1 %	
All-Cause Mortality (%)	31.2 %		0 %		100 %	
Cardiovascular Disease Mortality (%)	14.3 %		0 %		47.8 %	
Educational Attainment- Less than High School (%)	25.6 %		20.8 %		36.4 %	
Educational Attainment- High School (%)	49.2 %		51.4 %		44.1 %	
Educational Attainment- Greater than High School (%)	25.2 %		27.8 %		19.5 %	
Body Mass Index (kg/m^2^)	32.38 (6.58)	31.32 (17.10, 57.97)	32.67 (6.40)	31.61 (17.10, 57.97)	31.75 (6.93)	30.54 (17.54, 56.92)
Waist Hip Ratio	0.941 (0.082)	0.944 (0.436, 1.246)	0.937 (0.077)	0.939 (0.648, 1.234)	0.949 (0.091)	0.955 (0.436, 1.246)
Glucose (mg/dL)	147.9 (56.15)	135 (16, 463)	145.6 (51.17)	134 (16, 463)	152.9 (65.60)	140 (46, 436)
Glycated Hemoglobin (%)	7.60 (1.72)	7.20 (4.30, 18.30)	7.49 (1.61)	7.10 (4.60, 16.40)	7.84 (1.92)	7.60 (4.30, 18.30)
Diabetes Duration (years)	10.42 (7.15)	8 (0, 46)	9.32 (6.39)	7 (0, 46)	12.80 (8.09)	11 (1, 41)
Coronary Artery Calcified Plaque (mass score)	1856 (3335)	449.5 (0, 50415)	1328 (3038)	256.5 (0, 50415)	3048 (3657)	1632 (0, 22378)
Carotid Intima Media Thickness (mm)	0.683 (0.136)	0.659 (0.449, 1.569)	0.664 (0.127)	0.639 (0.449, 1.569)	0.723 (0.144)	0.700 (0.456, 1.316)
Total Cholesterol (mg/dL)	185.2 (43.42)	181 (65, 427)	184.5 (43.15)	180 (65, 427)	186.9 (44.04)	183 (70, 386)
HDL (mg/dL)	42.16 (11.89)	41 (8, 98)	42.46 (11.59)	41 (14, 94)	41.49 (12.52)	40 (8, 98)
Triglycerides (mg/dL)	208.9 (138.8)	176 (30, 1310)	206.0 (136.4)	173 (30, 1310)	215.3 (143.9)	183 (30, 1065)
LDL (mg/dL)	103.0 (32.6)	100 (12, 236)	102.2 (32.4)	100 (14, 207)	104.9 (33.1)	103 (12, 236)
Pulse Pressure (mmHg)	67.09 (16.93)	65.5 (28, 159)	65.53 (15.85)	64 (28, 124)	70.52 (18.67)	69.5 (31, 159)
Mean Arterial Pressure (mmHg)	95.05 (11.24)	94 (66.5, 154)	95.19 (10.33)	94 (66.5, 140.3)	94.75 (13.02)	93.17 (67.33, 154)
Estimated Glomerular Filtration Rate (ml/min/1.73 m^2^)	65.76 (18.38)	63.77 (8.91, 126.4)	67.95 (17.86)	65.43 (20.97, 122.2)	60.96 (18.62)	58.43 (8.91, 126.4)
Urine Albumin:creatinine Ratio (mg/g)	121.5 (530.5)	14.29 (0.48, 9449)	68.49 (390.8)	11.57 (0.48, 8878)	239.8 (741.5)	23.08 (0.78, 9449)
QT Interval (ms)	393.8 (33.14)	392.0 (270.1, 564.0)	393.2 (32.27)	392.0 (310.1, 564.0)	395.0 (34.93)	392.0 (270.1, 526.0)
High Blood Pressure Medications (%)	75.5 %		72.0 %		83.4 %	
Statin Use (%)	42.7 %		44.8 %		37.9 %	
Oral Hypoglycemic Medications (%)	78.8 %		79.0 %		78.4 %	
Insulin Use (%)	27.3 %		22.9 %		37.0 %	
C-reactive Protein (mg/dL)	0.611 (1.004)	0.296 (0.005, 12.73)	0.529 (0.793)	0.28 (0.005,9.573)	0.801 (1.355)	0.353 (0.008,12.73)
Biventricular Volume (ml)	384.5 (112.1)	375.6 (156.6, 884.5)	372.0 (100.7)	361.3 (156.6, 781.8)	411.8 (129.7)	397.2 (182.6, 884.5)

The univariate associations of demographic and clinical factors with all-cause and CVD mortality in the full EA T2D-affected cohort are shown in Table 2. Demographic and clinical factors were selected for model selection based on their associations with all-cause or CVD mortality (*p* < 0.25) (Table 2). Based on these univariate associations, model selection for all-cause mortality included HbA_1c_, CAC, pulse pressure, HDL, eGFR, UACR, diabetes duration, BMI, high blood pressure medication use, insulin use, current smoking, history of CVD, educational attainment (less than high school, high school, greater than high school), WHR, mean arterial pressure, and IMT. Model selection for CVD mortality included fasting glucose, HbA_1c_, CAC, pulse pressure, HDL, triglycerides, eGFR, UACR, QT interval, diabetes duration, BMI, high blood pressure medication use, insulin use, history of CVD, educational attainment, WHR, and IMT.

**Table 2:** Associations of demographic and clinical factors with all-cause and cardiovascular disease (CVD) mortality

	All-cause Mortality					CVD mortality				
Trait	HR	95 % HR CI		*p*-value	*n*	HR	95 % HR CI		*p*-value	*n*
Age (years)	1.86	1.62	2.13	<1 × 10^−16^	1022	1.74	1.41	2.13	1.40 × 10^−7^	1004
Female Sex (%)	0.68	0.54	0.85	8.83 × 10^−4^	1022	0.58	0.41	0.82	0.002	1004
Current Smoking (%)	1.44	1.11	1.88	0.006	1018	1.04	0.66	1.63	0.866	1000
Past Smoking (%)	0.99	0.79	1.23	0.896	1018	1.13	0.82	1.57	0.452	1000
History of Cardiovascular Disease (%)	1.91	1.52	2.41	3.86 × 10^−8^	1013	3.25	2.25	4.69	3.21 × 10^−10^	995
Educational attainment (3 levels)	0.68	0.58	0.81	1.73 × 10^−5^	1011	0.74	0.58	0.95	0.02	993
dy Mass Index (kg/m^2^)	0.87	0.77	0.99	0.033	1022	0.89	0.74	1.07	0.225	1004
Waist Hip Ratio	1.16	1.02	1.32	0.029	1012	1.2	1	1.45	0.049	994
Glucose (mg/dL)	1.04	0.92	1.17	0.512	1020	1.16	0.98	1.38	0.089	1002
Glycated Hemoglobin (%)	1.09	0.97	1.22	0.169	1016	1.23	1.06	1.43	0.008	998
Diabetes duration (years)	1.54	1.36	1.74	5.62 × 10^−12^	1005	1.7	1.41	2.04	2.22 × 10^−8^	987
Coronary Artery Calcified Plaque (mass score)	2.01	1.7	2.37	1.11 × 10^−16^	968	2.47	1.91	3.21	8.48 × 10^−12^	952
Carotid Intima Media Thickness (mm)	1.38	1.25	1.53	5.20 × 10^−10^	928	1.42	1.24	1.63	7.32 × 10^−7^	911
Total Cholesterol (mg/dL)	1.01	0.91	1.13	0.796	1003	1	0.86	1.17	1	986
HDL (mg/dL)	0.93	0.82	1.05	0.228	1003	0.87	0.72	1.04	0.133	986
Triglycerides (mg/dL)	1.01	0.9	1.14	0.841	1003	1.12	0.95	1.33	0.172	986
LDL (mg/dL)	1.05	0.94	1.17	0.413	928	0.93	0.79	1.1	0.396	912
Pulse Pressure (mmHg)	1.3	1.16	1.46	8.24 × 10^−6^	1019	1.35	1.13	1.61	7.71 × 10^−4^	1001
Mean Arterial Pressure (mmHg)	0.9	0.79	1.04	0.149	1019	0.95	0.76	1.2	0.685	1001
Estimated Glomerular Filtration Rate (ml/min/1.73 m^2^)	0.63	0.55	0.72	3.60 × 10^−11^	1021	0.63	0.51	0.78	2.75 × 10^−5^	1003
Urine Albumin:creatinine Ratio (mg/g)	1.56	1.42	1.72	<1 × 10^−16^	1000	1.76	1.54	2.01	<1 × 10^−16^	982
QT Interval (ms)	1.02	0.91	1.16	0.709	985	1.13	0.93	1.38	0.212	968
High Blood Pressure Medications (%)	1.72	1.28	2.3	2.63 × 10^−4^	1022	2.28	1.41	3.69	7.86 × 10^−4^	1004
Statin Use (%)	0.88	0.7	1.11	0.293	1020	1.02	0.72	1.46	0.91	1002
Oral Hypoglycemic Medications (%)	1.04	0.79	1.38	0.769	1022	1.02	0.67	1.54	0.942	1004
Insulin Use (%)	1.71	1.35	2.17	8.51 × 10^−6^	1022	1.84	1.32	2.58	3.55 × 10^−4^	1004

Relationships assessed using univariate Cox proportional hazards models. Hazards ratios (HRs) are for a one standard deviation change in the predictor (continuous variables) or change in group assignment (dichotomous variables). For medication use HRs, the HRs are for risk of mortality among those individuals using the given medication class. CI, Confidence Interval

The all-cause mortality model selected from the full sample included age, sex, CAC, UACR, insulin use, current smoking, and educational attainment (Table 3). Other than age, the most significant associations with all-cause mortality were for CAC (*p* = 2.03 × 10^−4^, hazard ratio (HR) = 1.48 for a standard deviation (SD) change) and UACR (*p* = 2.23 × 10^−8^, HR = 1.37 for a SD change). The CVD mortality model selected from the full sample included age, sex, CAC, UACR, triglycerides, and history of CVD (Table 3). Similar to the all-cause mortality analysis, other than age, the most significant associations with CVD mortality were for CAC (*p* = 0.001, HR = 1.71 for a SD change) and UACR (*p* = 1.99 × 10^−8^, HR = 1.51 for a SD change).

**Table 3:** Models selected for all-cause and cardiovascular disease (CVD) mortality in European Americans with type 2 diabetes

Trait	Hazard Ratio	95 % Hazard Ratio Confidence Interval		*p*-value
All-cause mortality				
Age	1.72	1.46	2.02	6.46 × 10^−11^
Female Sex	0.93	0.71	1.21	0.565
Urine Albumin:creatinine Ratio	1.37	1.23	1.53	2.23 × 10^−8^
Coronary Artery Calcified Plaque	1.48	1.20	1.81	2.03 × 10^−4^
Current Smoking	1.79	1.30	2.46	3.38 × 10^−4^
Insulin Use	1.50	1.16	1.93	0.002
Educational Attainment	0.76	0.65	0.91	0.002
CVD mortality				
Age	1.57	1.18	2.08	0.002
Female Sex	0.94	0.63	1.40	0.756
Urine Albumin:creatinine Ratio	1.51	1.31	1.74	1.99 × 10^−8^
Coronary Artery Calcified Plaque	1.71	1.23	2.38	0.001
Triglycerides	1.28	1.05	1.56	0.017
History of Cardiovascular Disease	1.59	1.03	2.46	0.036

Models were selected using backward elimination, forward selection, and stepwise selection. Age and sex were forced into all models. Hazards ratios (HRs) are for a one standard deviation change in the predictor (continuous variables) or change in group assignment (dichotomous variables). For medication use HRs, the HRs are for risk of mortality among those individuals using the given medication class

To confirm the validity of the most important predictors identified with model selection using the full DHS sample, a two-fold cross-validation approach was used, where the dataset was randomly split by family into roughly equal halves. The analysis for the full sample was repeated separately in each random dataset. In each random dataset, demographic and clinical factors were selected to include in model building based on their univariate associations with all-cause or CVD mortality (Additional file 1). Models were selected using forward, backward, and stepwise selections for all-cause and CVD mortality in random datasets 1 and 2 (Additional files 2 and 3). As the associated predictors selected from each dataset differed slightly, a combined model including factors selected in the final model for each dataset was created for both all-cause and CVD mortality. Therefore, from this two-fold cross-validation approach, the all-cause mortality model included age, sex, CAC, UACR, diabetes duration, current smoking, educational attainment, insulin use, and WHR, while the CVD mortality model included age, sex, CAC, UACR, history of CVD events, and diabetes duration. The factors selected are similar to those observed using the full sample, which shows that the results are stable with no evidence of overfitting. This full model was fitted in both random datasets, with a basic model limited to age and sex also fitted as a comparison to the full model.

Predicted values were then derived in each randomly selected dataset using regression coefficients derived from fitting the model in the opposite dataset, allowing calculation of average AUC for each dataset (Table 4), both for the full model derived using the two-fold cross-validation approach and a basic model including only age and sex. The full model significantly improved prediction of both all-cause and CVD mortality (*p* < 0.05) in both random datasets (Table 4). An estimate of AUC for the whole EA cohort was also derived by fitting the models derived using the two-fold cross-validation approach in the whole sample, with an AUC of 0.788 for the full all-cause mortality model in the whole sample, compared to 0.699 for a basic model containing only age and sex (*p* = 6.50 × 10^−9^ for improved prediction using the full model). Similarly, the full CVD mortality model had an AUC of 0.764, compared to 0.652 for a basic model containing only age and sex (*p* = 4.14 × 10^−7^ for improved prediction using the full model) (Table 4).

**Table 4:** Average area under the receiver operating characteristic curve (AUC) for the all-cause and cardiovascular disease (CVD) mortality models

Data	Outcome	Model	AUC	95 % Confidence Interval	*p*-value
Random 1	All-cause mortality	Basic	0.697	(0.644, 0.749)	0.011
	All-cause mortality	Full	0.759	(0.710, 0.807)	
	CVD mortality	Basic	0.658	(0.590, 0.726)	0.002
	CVD mortality	Full	0.764	(0.705, 0.823)	
Random 2	All-cause mortality	Basic	0.693	(0.638, 0.747)	0.0001
	All-cause mortality	Full	0.777	(0.732, 0.823)	
	CVD mortality	Basic	0.614	(0.534, 0.694)	0.0003
	CVD mortality	Full	0.725	(0.661, 0.789)	
Full Cohort	All-cause mortality	Basic	0.699	(0.661, 0.737)	6.50 × 10^−9^
	All-cause mortality	Full	0.788	(0.756, 0.821)	
	CVD mortality	Basic	0.652	(0.600, 0.704)	4.14 × 10^−7^
	CVD mortality	Full	0.764	(0.721, 0.807)	

These models were derived using a two-fold cross-validation approach in each randomly selected dataset and in the full cohort. Basic models included age and sex only. Full models for all-cause mortality included age, sex, coronary artery calcified plaque, urine albumin:creatinine ratio, diabetes duration, current smoking, educational attainment, insulin use, and waist hip ratio. Full models for CVD mortality included age, sex, coronary artery calcified plaque, urine albumin:creatinine ratio, history of CVD events, and diabetes duration. *P*-values for comparing the predictive power of the basic model with the full model are listed

Factors which were predictors of mortality in the DHS in previous analyses, such as CRP [4] and biventricular volume [5], were not included in the main model selection due to lower sample size (*n* = 848 for CRP, *n* = 771 for biventricular volume). When adding biventricular volume to the full all-cause and CVD mortality models selected using a two-fold cross-validation approach, biventricular volume was nominally associated with increased all-cause mortality risk (HR = 1.21 for a SD change, *p* = 0.053) (Table 5) and more strongly associated with CVD mortality risk (HR = 1.52 for a SD change, *p* = 0.002) (Table 6). In contrast, CRP was a significant predictor when added to the all-cause mortality model (HR = 1.30 for a SD change, *p* = 0.001) (Table 5), but not the CVD mortality model (HR = 1.19 for a SD change, *p* = 0.167) (Table 6).

**Table 5:** Addition of C-reactive protein and biventricular volume to model selected for all-cause mortality

Trait	Hazard Ratio	95 % Hazard Ratio Confidence Interval		*p*-value	Trait	Hazard Ratio	95 % Hazard Ratio Confidence Interval		*p*-value	Trait	Hazard Ratio	95 % Hazard Ratio Confidence Interval		*p*-value
Age	1.67	1.42	1.96	3.68 × 10^−10^	Age	1.80	1.52	2.13	6.54 × 10^−12^	Age	1.81	1.53	2.15	9.65 × 10^−12^
Female Sex	0.92	0.70	1.22	0.581	Female Sex	0.86	0.62	1.19	0.356	Female Sex	1.24	0.85	1.81	0.269
Coronary Artery Calcified Plaque	1.50	1.24	1.81	3.32 × 10^−5^	Coronary Artery Calcified Plaque	1.44	1.17	1.77	0.001	Coronary Artery Calcified Plaque	1.45	1.15	1.82	0.001
Urine Albumin: creatinine Ratio	1.35	1.21	1.51	1.10 × 10^−7^	Urine Albumin: creatinine Ratio	1.35	1.20	1.52	7.85 × 10^−7^	Urine Albumin: creatinine Ratio	1.32	1.17	1.49	8.08 × 10^−6^
Diabetes Duration	1.10	0.96	1.26	0.173	Diabetes Duration	1.05	0.91	1.21	0.505	Diabetes Duration	1.09	0.94	1.25	0.261
Current Smoking	1.70	1.25	2.31	7.38 × 10^−4^	Current Smoking	1.63	1.16	2.28	0.005	Current Smoking	2.00	1.42	2.83	8.46 × 10^−5^
Educational Attainment	0.79	0.67	0.93	0.004	Educational Attainment	0.79	0.66	0.94	0.010	Educational Attainment	0.78	0.65	0.94	0.008
Insulin Use	1.40	1.07	1.84	0.015	Insulin Use	1.49	1.11	2.00	0.008	Insulin Use	1.39	1.03	1.86	0.029
Waist Hip Ratio	1.01	0.86	1.19	0.913	Waist Hip Ratio	0.97	0.81	1.17	0.765	Waist Hip Ratio	1.01	0.86	1.20	0.868
					C-reactive Protein	1.30	1.11	1.52	0.001	Biventricular Volume	1.21	1.00	1.48	0.053

The all-cause mortality model was selected using a two-fold cross-validation approach in European American participants with type 2 diabetes. Hazards ratios (HRs) are for a one standard deviation change in the predictor (continuous variables) or change in group assignment (dichotomous variables). For medication use HRs, the HRs are for risk of mortality among those individuals using the given medication class

**Table 6:** Addition of C-reactive protein and biventricular volume to model selected for cardiovascular disease mortality

Trait	Hazard Ratio	95 % Hazard Ratio Confidence Interval		*p*-value	Trait	Hazard Ratio	95 % Hazard Ratio Confidence Interval		*p*-value	Trait	Hazard Ratio	95 % Hazard Ratio Confidence Interval		*p*-value
Age	1.36	1.08	1.73	0.010	Age	1.63	1.27	2.10	1.20 × 10^−4^	Age	1.75	1.36	2.27	1.66 × 10^−5^
Female Sex	0.98	0.68	1.42	0.929	Female Sex	1.02	0.67	1.55	0.942	Female Sex	1.52	0.90	2.55	0.116
Coronary Artery Calcified Plaque	1.67	1.24	2.26	7.18 × 10^−4^	Coronary Artery Calcified Plaque	1.60	1.15	2.23	0.005	Coronary Artery Calcified Plaque	1.40	0.98	2.00	0.067
Urine Albumin: creatinine Ratio	1.52	1.32	1.75	7.59 × 10^−9^	Urine Albumin: creatinine Ratio	1.57	1.35	1.83	7.31 × 10^−9^	Urine Albumin: creatinine Ratio	1.45	1.24	1.70	3.29 × 10^−6^
History of Cardiovascular Disease	1.69	1.11	2.57	0.014	History of Cardiovascular Disease	1.52	0.95	2.43	0.084	History of Cardiovascular Disease	1.55	0.98	2.45	0.063
Diabetes Duration	1.20	0.98	1.46	0.082	Diabetes Duration	1.04	0.84	1.28	0.736	Diabetes Duration	1.09	0.88	1.36	0.435
					C-reactive Protein	1.19	0.93	1.51	0.167	Biventricular Volume	1.52	1.16	1.98	0.002

The cardiovascular disease mortality model was selected using a two-fold cross-validation approach in European Americans with type 2 diabetes. Hazards ratios (HRs) are for a one standard deviation change in the predictor (continuous variables) or change in group assignment (dichotomous variables). For medication use HRs, the HRs are for risk of mortality among those individuals using the given medication class

## Discussion

This set of DHS analyses highlights demographic and clinical factors independently predictive of mortality in EAs with T2D. Most prior mortality model analyses in individuals with T2D have included a more limited set of clinical and demographic factors, for example BMI, lipids, medications, glycemic control, and eGFR, compared to those available in the DHS. As such, the literature does not fully address which factors may predict all-cause and CVD mortality by considering novel predictors, particularly CAC, in individuals with T2D. All the clinical and demographic factors included in model selection can be hypothesized to contribute to mortality. Our analysis shows, out of many mortality associated predictors, which ones are the most important to consider in T2D-affected individuals using a statistical model selection approach. While results from model selection for all-cause and CVD mortality differed slightly between model selection using the full cohort and model selection using a two-fold cross-validation approach, the key factors selected, notably CAC and UACR, did not differ. This demonstrates the validity of model selection results. Other factors selected using the full sample, such as current smoking, insulin use, and educational attainment with all-cause mortality and history of cardiovascular disease with CVD mortality, were also included in the models selected using a two-fold cross-validation approach. This indicates results from the model selection are stable and reveals important factors to consider in future analyses of mortality in community-based cohorts of T2D-affected individuals.

The two factors most consistently associated with all-cause and CVD mortality were CAC and albuminuria. Prior studies in T2D have demonstrated striking associations of UACR with renal end-points, CVD events, CVD mortality, and all-cause mortality [18–20]. Prior mortality analyses with shorter follow-up in the full DHS cohort with and without T2D (83.7 % T2D-affected) also found UACR to be associated with all-cause and CVD mortality, independent from CAC and other CVD risk factors such as age, sex, T2D affection status, BMI, current smoking, hypertension, dyslipidemia, renin-angiotensin system blocking medications, and prior CVD [7]. As in prior analyses of individuals with T2D [19], UACR was a stronger predictor of CVD mortality than eGFR in our models. UACR is a marker of generalized endothelial dysfunction, more so than kidney disease specifically, which is better reflected by changes in eGFR. UACR was not assessed in some analyses building mortality prediction models in T2D [11, 12], but it was selected as a significant predictor of elevated all-cause mortality in the Hong Kong Diabetes Registry cohort [13] and the Gargano Mortality Study [14]. The incorporation of CAC significantly improves mortality risk prediction, which is expected. CAC is a strong independent predictor of CVD events and mortality in the general population and in T2D [2, 3, 21–27], with individuals affected by diabetes tending to have higher CAC [28]. Recent model building using participants with T2D from the Multi-Ethnic Study of Atherosclerosis (MESA) and the Heinz Nixdorf Recall Study found that using CAC improved risk prediction for incident CVD events, an important contributor to mortality risk in those with T2D, above the Framingham or United Kingdom Prospective Diabetes study risk models which include more conventional risk factors such as age, sex, systolic blood pressure, HbA_1c_, and lipid levels [9]. Notably, carotid IMT, another novel predictor of CVD risk in T2D, was not selected for in these CVD event prediction models in T2D [9], consistent with our finding that IMT was not an independent predictor of mortality in the DHS and previous literature indicating that CAC provides superior risk prediction and reclassification compared to carotid IMT [29, 30].

Demographic factors including current smoking, insulin use, and educational attainment were also associated with all-cause mortality in the DHS. Current smoking [11, 12] and use of insulin [11–14] have been previously included in prediction models as associated with elevated mortality risk in analyses of individuals with T2D. Lower educational attainment has also been associated with elevated mortality risk in prior assessments of T2D, although this was not always included in final selected models, potentially due to the inclusion of correlated variables such as income that were not used in our analysis [12]. History of CVD events being associated with CVD mortality is not surprising, given the elevated risk of secondary CVD events in those with established CVD. History of CVD is strongly associated with CAC burden (*p* < 1 × 10^−16^) but is still an independent predictor of CVD, but not all-cause, mortality.

Additional factors not included in the primary model building analysis due to reduced sample size may also be important to mortality risk prediction in T2D. Previous analysis in the DHS with shorter mortality follow-up time suggested that biventricular volume significantly increased AUC for prediction of all-cause and CVD mortality versus a model including age, sex, and CAC alone [5]. This is substantiated by data from this analysis, with biventricular volume improving prediction of CVD mortality (Table 6). Biventricular volume can be derived from the same CT scans used to evaluate CAC, but it appears to be predictive of CVD mortality independent of CAC burden, so this may be an important factor to include in future analyses of mortality in T2D. CRP may also need to be considered in future analyses. In a previously published analysis from the DHS, each one unit increase in log transformed CRP was associated with a 1.5-fold increase in risk for all-cause mortality (HR 1.54; 95 % CI 1.33–1.77) [4]. Similar results were observed, though with a slightly attenuated HR, when CRP was added to our full all-cause mortality model from this analysis (Table 5). The lack of association with CVD mortality for CRP may not be surprising, as some studies have cast doubt on the causal role of CRP in CVD events [31].

Recent work in the DHS highlighted demographic and clinical factors associated with mortality in individuals with T2D at very high CVD risk based on a CAC score >1000, with, for example, higher HbA_1c_, longer diabetes duration, reduced kidney function, reduced use of statins, and higher CRP associated with higher mortality [32]. We attempted to perform model selection in only T2D affected individuals with CAC >1000 using similar methods to the analysis in the full T2D-affected DHS sample (data not shown). Models were not perfectly concordant between forward, backward, and stepwise selection, not surprisingly given the reduced sample size (*n* = 371), but selected models all included CAC, UACR, and HbA_1c_ as contributors to elevated risk of all-cause and CVD mortality in this very high risk sample, similar to the results in the full T2D-affected cohort with the exception of HbA_1c_, which was not selected in the full T2D-affected cohort but appears to be a more important predictor in the CAC > 1000 subgroup.

Limitations of the current analysis include the lack of a replication cohort with similar mortality data and demographic and clinical factors assessed, necessitating use of a two-fold cross-validation approach. There are, however, few studies with the same breadth of data in a population-based sample of individuals with T2D. The latter, we believe, is an important feature of the DHS since it likely reflects what actually influences mortality in the community, as opposed to in a clinical trial. Previous analyses have been performed in cohorts with different recruitment strategies than the DHS, for example population-based cohorts in Hong Kong [13] and Italy [14], or cohorts extracted from electronic medical record data whose diabetes was not as well characterized as the DHS and with exclusions based on medication use [11]. None of these cohorts had data on all the novel measures of CVD risk available in the DHS. Length of follow-up, over 9 years on average, was also greater than in previous analyses [11–14], as was duration of T2D in study participants [13, 14]. This long follow-up does mean that some clinical guidelines and practices, for example recommendations for statin use in all individuals age 40–75 affected by diabetes [33], have changed since the DHS was initiated and may influence future mortality risk. Data for some demographic and clinical factors which predicted all-cause mortality in prior analyses in T2D, such as cancer [13] or Charlson index, a measure of comorbidity burden [12], were not available in the DHS, while some factors available in the DHS, notably CAC, were not used in prior model selection, making direct comparisons of mortality models derived in T2D-affected individuals difficult.

Additional analytic limitations include the relatively small number of events, in particular for CVD mortality (14.3 % of the cohort), which necessitated use of only two-fold cross validation, as opposed to an analysis strategy splitting the cohort by family into a greater number of small random samples. Further validation of our risk prediction models in additional cohorts of T2D-affected patients would be necessary prior to clinical applications. While these results highlight factors that may be important for mortality risk assessment in the T2D-affected population, causal relationships are uncertain. The current analyses were limited to individuals of EA descent, since levels of CAC differ from those in African Americans; different factors may impact mortality risk in other ethnic groups.

## Conclusions

The present results demonstrate the importance of assessing UACR and CAC in particular for prediction of CVD and all-cause mortality. Not all patients with T2D are at equal risk of poor health outcomes and mortality, and more comprehensive assessment in research and clinical settings of these predictors of mortality, particularly CAC, in T2D-affected individuals is needed.

## Additional files

Additional file 1:
**Associations of demographic and clinical factors with all-cause and cardiovascular disease mortality in two randomly selected datasets from European Americans with type 2 diabetes.** Associations with all-cause and cardiovascular disease mortality were assessed using univariate Cox proportional hazards models. Hazards ratios (HRs) are for a one standard deviation change in the predictor (continuous variables) or change in group assignment (dichotomous variables). For medication use HRs, the HRs are for risk of mortality among those individuals using the given medication class. Additional file 2:
**Model selected for all-cause mortality using backward elimination, forward selection, and stepwise selection in two randomly selected datasets from European American participants with type 2 diabetes.** Age and sex were forced into all models. Hazards ratios (HRs) are for a one standard deviation change in the predictor (continuous variables) or change in group assignment (dichotomous variables). For medication use HRs, the HRs are for risk of mortality among those individuals using the given medication class. Additional file 3:
**Model selected for cardiovascular disease mortality using backward elimination, forward selection, and stepwise selection in two randomly selected datasets from European Americans with type 2 diabetes.** Age and sex were forced into all models. Hazards ratios (HRs) are for a one standard deviation change in the predictor (continuous variables) or change in group assignment (dichotomous variables). For medication use HRs, the HRs are for risk of mortality among those individuals using the given medication class.
