# Supplementary material for: Predictors of all-cause and cardiovascular disease mortality in type 2 diabetes: Diabetes Heart Study
Source: Diabetol Metab Syndr. 2015 Jun 28;7:58. doi: 10.1186/s13098-015-0055-y (PMC4490739; doi:10.1186/s13098-015-0055-y)
Supplement: Additional file 1: — Associations of demographic and clinical factors with all-cause and cardiovascular disease mortality in two randomly selected datasets from European Americans with type 2 diabetes. Associations with all-cause and cardiovascular disease mortality were assessed using univariate Cox proportional hazards models. Hazards ratios (HRs) are for a one standard deviation change in the predictor (continuous variables) or change in group assignment (dichotomous variables). For medication use HRs, the HRs are for risk of mortality among those individuals using the given medication class. [file 13098_2015_55_MOESM1_ESM.pdf]

Additional file 1. Associations of demographic and clinical factors with all-cause and cardiovascular disease mortality in two randomly selected datasets from European Americans with type 2 diabetes. Associations with all-cause and cardiovascular disease mortality were assessed using univariate Cox proportional hazards models. Hazards ratios (HRs) are for a one standard deviation change in the predictor (continuous variables) or change in group assignment (dichotomous variables). For medication use HRs, the HRs are for risk of mortality among those individuals using the given medication class.

	**Random dataset 1**				**Random dataset 2**			
	**All-cause mortality**		**Cardiovascular disease mortality**		**All-cause mortality**		**Cardiovascular disease mortality**	
**Trait**	**Hazard Ratio**	**p-value**	**Hazard Ratio**	**p-value**	**Hazard Ratio**	**p-value**	**Hazard Ratio**	**p-value**
**Age (years)**	1.90	7.09×10^{-11}	1.96	2.78×10^{-6}	1.81	2.44×10^{-9}	1.50	0.007
**Female Sex (%)**	0.76	0.091	0.65	0.057	0.59	0.002	0.50	0.015
**Current Smoking (%)**	1.20	0.354	0.68	0.277	1.75	0.002	1.57	0.122
**Past Smoking (%)**	1.02	0.882	1.26	0.317	0.95	0.722	1.00	0.997
**History of Cardiovascular Disease (%)**	1.78	3.69×10^{-4}	3.43	2.50×10^{-6}	2.04	4.48×10^{-5}	2.94	7.09×10^{-5}
**Educational Attainment (3 levels)**	0.71	0.006	0.69	0.029	0.66	5.58×10^{-4}	0.84	0.362
**Body Mass Index (kg/m^2)**	0.81	0.017	0.78	0.056	0.94	0.491	1.03	0.809
**Waist Hip Ratio**	1.23	0.015	1.25	0.055	1.05	0.626	1.11	0.460
**Glucose (mg/dL)**	1.04	0.680	1.14	0.319	1.04	0.611	1.19	0.117
**Glycated Hemoglobin (%)**	1.06	0.492	1.16	0.163	1.12	0.200	1.32	0.010

**Diabetes Duration (years)**	1.55	4.60×10^{-7}	1.69	2.79×10^{-5}	1.53	2.84×10^{-6}	1.70	1.83×10^{-4}
**Coronary Artery Calcified Plaque (mass score)**	1.82	4.46×10^{-7}	2.59	8.60×10^{-8}	2.23	1.12×10^{-11}	2.38	1.84×10^{-5}
**Carotid Intima Media Thickness (mm)**	1.42	2.16×10^{-6}	1.54	7.72×10^{-6}	1.35	1.05×10^{-5}	1.33	0.003
**Total Cholesterol (mg/dL)**	0.98	0.824	0.99	0.953	1.05	0.508	1.01	0.922
**HDL (mg/dL)**	1.00	0.962	0.92	0.479	0.83	0.057	0.80	0.123
**Triglycerides (mg/dL)**	0.96	0.618	1.09	0.457	1.08	0.365	1.16	0.198
**LDL (mg/dL)**	1.02	0.783	0.93	0.564	1.07	0.340	0.93	0.532
**Pulse pressure (mmHg)**	1.37	1.45×10^{-5}	1.41	9.17×10^{-4}	1.21	0.042	1.26	0.132
**Mean Arterial Pressure (mmHg)**	0.91	0.369	0.92	0.601	0.89	0.240	0.99	0.944
**Estimated Glomerular Filtration Rate (ml/min/1.73m^2)**	0.65	9.16×10^{-6}	0.57	1.55×10^{-4}	0.61	3.82×10^{-7}	0.71	0.032
**Urine Albumin: creatinine Ratio (mg/g)**	1.50	1.96×10^{-10}	1.73	1.57×10^{-12}	1.64	1.33×10^{-10}	1.78	2.89×10^{-6}
**QT interval (ms)**	1.00	0.976	1.14	0.285	1.04	0.716	1.10	0.552
**High Blood Pressure Medications (%)**	1.66	0.014	2.11	0.021	1.81	0.004	2.57	0.011
**Statin Use (%)**	0.87	0.392	1.01	0.961	0.90	0.526	1.03	0.914

**Oral Hypoglycemic Medications (%)**	0.91	0.590	0.88	0.609	1.28	0.308	1.32	0.464
**Insulin Use (%)**	1.93	4.04×10^{-5}	2.31	1.22×10^{-4}	1.46	0.040	1.32	0.340

For random dataset 1, coronary artery calcified plaque, pulse pressure, estimated glomerular filtration rate, urine albumin:creatinine ratio, diabetes duration, body mass index, high blood pressure medications, insulin use, history of cardiovascular disease, educational attainment, waist hip ratio, and carotid intima media thickness were included in model selection for all-cause mortality; for cardiovascular disease mortality, glycated hemoglobin, coronary artery calcified plaque, pulse pressure, estimated glomerular filtration rate, urine albumin:creatinine ratio, diabetes duration, body mass index, high blood pressure medications, insulin use, history of cardiovascular disease, educational attainment, waist hip ratio, and carotid intima media thickness were included in model selection.

For random dataset 2, glycated hemoglobin, coronary artery calcified plaque, pulse pressure, HDL, estimated glomerular filtration rate, urine albumin:creatinine ratio, diabetes duration, high blood pressure medications, insulin use, current smoking, history of cardiovascular disease, educational attainment, carotid intima media thickness, and mean arterial pressure were included in model selection for all-cause mortality; for cardiovascular disease mortality, fasting glucose, glycated hemoglobin, coronary artery calcified plaque, pulse pressure, HDL, triglycerides, estimated glomerular filtration rate, urine albumin:creatinine ratio, diabetes duration, high blood pressure medications, current smoking, history of cardiovascular disease, and carotid intima media thickness were included in model selection.

Age and sex were included in all models.
