# Supplementary material for: Predictors of all-cause and cardiovascular disease mortality in type 2 diabetes: Diabetes Heart Study
Source: Diabetol Metab Syndr. 2015 Jun 28;7:58. doi: 10.1186/s13098-015-0055-y (PMC4490739; doi:10.1186/s13098-015-0055-y)
Supplement: Additional file 3: — Model selected for cardiovascular disease mortality using backward elimination, forward selection, and stepwise selection in two randomly selected datasets from European Americans with type 2 diabetes. Age and sex were forced into all models. Hazards ratios (HRs) are for a one standard deviation change in the predictor (continuous variables) or change in group assignment (dichotomous variables). For medication use HRs, the HRs are for risk of mortality among those individuals using the given medication class. [file 13098_2015_55_MOESM3_ESM.pdf]

Additional file 3. Model selected for cardiovascular disease mortality using backward elimination, forward selection, and stepwise selection in two randomly selected datasets from European Americans with type 2 diabetes. Age and sex were forced into all models. Hazards ratios (HRs) are for a one standard deviation change in the predictor (continuous variables) or change in group assignment (dichotomous variables). For medication use HRs, the HRs are for risk of mortality among those individuals using the given medication class.

**Random dataset 1**					**Random dataset 2**				
**Trait**	**Hazard Ratio**	**95% Hazard Ratio Confidence Interval**		**p-value**	**Trait**	**Hazard Ratio**	**95% Hazard Ratio Confidence Interval**		**p-value**
**Age**	1.73	1.23	2.42	0.002	**Age**	1.03	0.71	1.50	0.874
**Female Sex**	1.10	0.66	1.84	0.711	**Female Sex**	0.85	0.46	1.58	0.607
**Coronary Artery Calcified Plaque**	1.64	1.06	2.54	0.027	**Coronary Artery Calcified Plaque**	2.00	1.31	3.03	0.001
**Urine Albumin: creatinine Ratio**	1.59	1.35	1.86	1.74×10^{-8}	**Urine Albumin: creatinine Ratio**	1.59	1.20	2.10	0.001
**History of Cardiovascular Disease**	1.83	1.04	3.23	0.036	**Diabetes Duration**	1.50	1.09	2.07	0.013
